# Development and external validation of the multichannel deep learning model based on unenhanced CT for differentiating fat-poor angiomyolipoma from renal cell carcinoma: a two-center retrospective study

**DOI:** 10.1007/s00432-023-05339-0

**Published:** 2023-09-06

**Authors:** Haohua Yao, Li Tian, Xi Liu, Shurong Li, Yuhang Chen, Jiazheng Cao, Zhiling Zhang, Zhenhua Chen, Zihao Feng, Quanhui Xu, Jiangquan Zhu, Yinghan Wang, Yan Guo, Wei Chen, Caixia Li, Peixing Li, Huanjun Wang, Junhang Luo

**Affiliations:** 1 Department of Urology, The First Affiliated Hospital, Sun Yat-Sen University, Guangzhou, China; 2 Department of Urology, Guangdong Provincial People’s Hospital, Guangdong Academy of Medical Sciences, Guangzhou, China; 3 Department of Medical Imaging, State Key Laboratory of Oncology in South China, Collaborative Innovation Center for Cancer Medicine, Sun Yat-Sen University Cancer Center, Guangzhou, China; 4 Department of Radiology, The First Affiliated Hospital, Sun Yat-Sen University, Guangzhou, China; 5 Department of Urology, Jiangmen Central Hospital, Jiangmen, China; 6 Department of Urology, State Key Laboratory of Oncology in South China, Collaborative Innovation Center for Cancer Medicine, Sun Yat-Sen University Cancer Center, Guangzhou, China; 7 School of Mathematics and Computational Science, Sun Yat-Sen University, Guangzhou, China

**Keywords:** Renal cell carcinoma, Fat-poor angiomyolipoma, Urology, Deep learning, Computed tomography

## Abstract

**Purpose:**

There are undetectable levels of fat in fat-poor angiomyolipoma. Thus, it is often misdiagnosed as renal cell carcinoma. We aimed to develop and evaluate a multichannel deep learning model for differentiating fat-poor angiomyolipoma (fp-AML) from renal cell carcinoma (RCC).

**Methods:**

This two-center retrospective study included 320 patients from the First Affiliated Hospital of Sun Yat-Sen University (FAHSYSU) and 132 patients from the Sun Yat-Sen University Cancer Center (SYSUCC). Data from patients at FAHSYSU were divided into a development dataset (n = 267) and a hold-out dataset (n = 53). The development dataset was used to obtain the optimal combination of CT modality and input channel. The hold-out dataset and SYSUCC dataset were used for independent internal and external validation, respectively.

**Results:**

In the development phase, models trained on unenhanced CT images performed significantly better than those trained on enhanced CT images based on the fivefold cross-validation. The best patient-level performance, with an average area under the receiver operating characteristic curve (AUC) of 0.951 ± 0.026 (mean ± SD), was achieved using the “unenhanced CT and 7-channel” model, which was finally selected as the optimal model. In the independent internal and external validation, AUCs of 0.966 (95% CI 0.919–1.000) and 0.898 (95% CI 0.824–0.972), respectively, were obtained using the optimal model. In addition, the performance of this model was better on large tumors (≥ 40 mm) in both internal and external validation.

**Conclusion:**

The promising results suggest that our multichannel deep learning classifier based on unenhanced whole-tumor CT images is a highly useful tool for differentiating fp-AML from RCC.

## Introduction

Renal angiomyolipoma (AML) is a form of benign solid tumor that is composed of fat, smooth muscle, and abnormal blood vessels in varying proportions. Because fat can show negative attenuation values on unenhanced computed tomography (CT) images, AML can be accurately diagnosed by detecting fat within tumors (Nelson and Sanda 2002; Jinzaki et al. 2014). However, fat-poor angiomyolipoma (fp-AML), a special type of AML, contains undetectable levels of fat or may be devoid of fat altogether, and is often misdiagnosed as renal cell carcinoma (RCC) (Fujii et al. 2008; Takahashi and Kawashima 2012; Jinzaki et al. 2014; Park 2017).

In practice, patients with fp-AML are always treated as RCC. Some patients with fp-AML that did not require nephrectomy were misdiagnosed as RCC and underwent radical nephrectomy; some patients with small fp-AML that did not require surgery underwent partial nephrectomy. A report from the Cleveland Clinic suggested that 55% of 219 patients with AML who underwent surgery were suspected to have RCC by the preoperative imaging examination (Lane et al. 2008). Patients with fp-AML could avoid unnecessary surgery, especially radical nephrectomy, if an accurate diagnosis can be obtained prior to surgery (Schachter et al. 2007; Campbell et al. 2021). Consequently, there is an urgent need to develop a novel strategy to accurately identify fp-AML before surgery.

Artificial intelligence (AI), including machine learning and deep learning, has become the focus of the medical field to assist diagnosis and provide clinical decision support (Oh and Jung 2004; Schmidhuber 2015; Tandel et al. 2019; Rezaeijo et al. 2022; Taghizadeh et al. 2022). Currently, AI has been increasingly applied in the analysis of medical images, and their potential has been demonstrated not only for disease screening (Long et al. 2017; Xia et al. 2018; Fu et al. 2020; Jahangirimehr et al. 2022), but also for the diagnosis and treatment of difficult cases (Anthimopoulos et al. 2016; Lu et al. 2018; Kavur et al. 2020; Castillo et al. 2022; Cui et al. 2022; Salmanpour et al. 2023). However, there are few studies using AI to assist in the identification of fp-AML and RCC (Hodgdon et al. 2015; Lee et al. 2017, 2018; Feng et al. 2018; Cui et al. 2019; Yang et al. 2020). Reviewing these previous studies, they have limitations such as small sample size, low accuracy, or lack of external validation.

The purpose of this study was to develop a multichannel deep learning model, which is trained using CT images of whole tumors, to classify fp-AML and three common pathological subtypes of RCC: clear cell renal cell carcinoma (ccRCC), papillary renal cell carcinoma (pRCC), and chromophobe renal cell carcinoma (chRCC).

## Methods

### Patient cohort

**Fig. 1:**
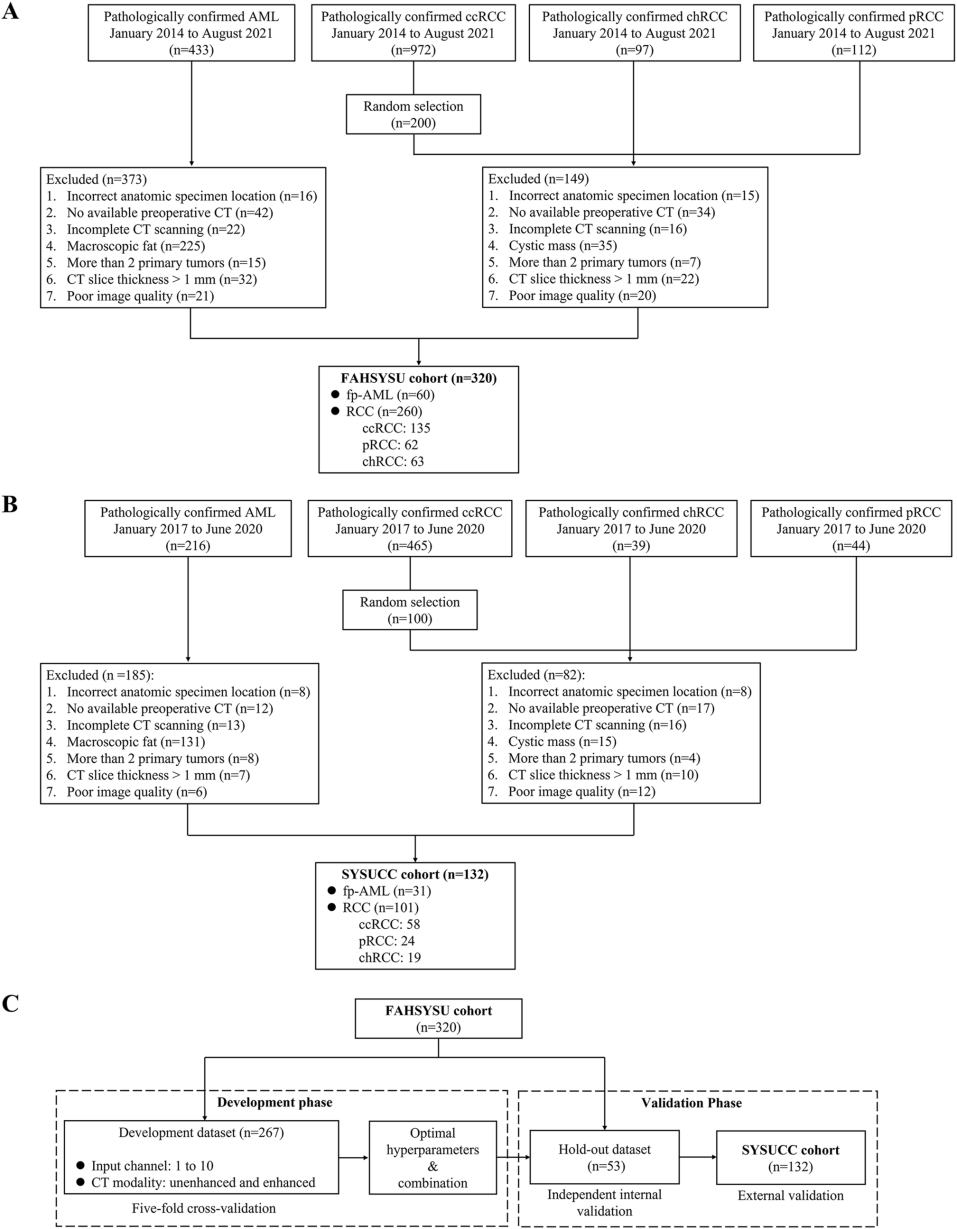
Flow chart of patient recruitment for the First Affiliated Hospital of Sun Yat-Sen University cohort (**a**) and Sun Yat-Sen University Cancer Center cohort (**b**), and the design for model development and validation (**c**)

We reviewed the medical records of patients with solid renal masses that were histologically diagnosed as AML, ccRCC, pRCC and chRCC at the First Affiliated Hospital of Sun Yat-Sen University (FAHSYSU) from January 2014 to August 2021 and at the Sun Yat-Sen University Cancer Center (SYSUCC) from January 2017 to June 2020. The data imbalance between patients with ccRCC and patients with the other pathological categories might affect the training of the model. Thus, data from the patients with ccRCC at both institutions were randomly downsampled by approximately 20%. Patients were excluded using the following criteria: (1) incorrect anatomic specimen location; (2) no available preoperative CT; (3) incomplete 4-phase CT scanning (unenhanced, corticomedullary, nephrographic, and excretory phases); (4) CT slice thickness > 1 mm; (5) poor image quality (such as motion artifacts or metal artifacts); and (6) more than 2 primary tumors. For the fp-AML group, patients with macroscopic fat within the tumor were excluded. For the RCC group, patients were excluded if the target lesion was primarily cystic. Finally, 60 fp-AML patients and 260 RCC patients (ccRCC, 135; pRCC, 62; chRCC, 63) from FAHSYSU and 31 fp-AML patients and 101 RCC patients (ccRCC, 58; pRCC, 24; chRCC, 19) from SYSUCC were enrolled in this study (Fig. 1a, b). The study was approved by the Ethics Committee of the First Affiliated Hospital of Sun Yat-Sen University (IIT-2022-678), and the requirement for individual consent for this retrospective analysis was waived.

### CT image preprocessing and labeling

All patients underwent CT examination using multi-slice spiral CT scanners (FAHSYSU: Aquilion 64, Toshiba, Tokyo, Japan; SYSUCC: Somatom Force, Siemens Healthineers, Forchheim, Germany). The unenhanced and corticomedullary enhanced CT slices from each patient were downloaded in digital imaging and communications in medicine (DICOM) format and converted to joint photographic experts group (JPEG) data with a resolution of 512 × 512 and a window of 40 × 300 (level × width) before labeling. CT slices without a target renal mass were excluded.

We cropped out the region of interest (ROI), a rectangular box, at the tumor location in each CT image, ensuring that the border of the rectangular box was close to the tumor. The boundary of the rectangular box was determined by two experienced radiologists, and a third radiologist was consulted in the case of disagreement. Once the boundary of the rectangle was determined, the image was assigned an ID, and the coordinates of the rectangular box and the pathological type of the tumor were recorded.

### Deep learning model development

The FAHSYSU dataset was divided into a development dataset (n = 267) and a hold-out dataset (n = 53). The development dataset was used to evaluate the model performance with different numbers of input channels and different CT modalities. The hold-out dataset was used for independent internal validation (Fig. 1c).

Our deep learning model was an end-to-end multichannel convolutional neural network (CNN) based on Xception architecture (Chollet 2017). We established 10 models with different numbers of input channels, from 1 to 10 channels. These models with different numbers of input channels were trained on enhanced and unenhanced whole-tumor CT images. Hence, there were 20 combinations of input channels and CT modalities. Fivefold cross-validation was used for training and validation for each combination. Here, the development dataset was split into five partitions before training, keeping the fp-AML and RCC labels balanced between partitions. In each iteration of cross-validation, one partition was utilized for validation, while the other four partitions were used for model training. Training and validation sets were always split on the patient level so that no CT slices from the same patient were ever part of a training set and a validation set.

**Fig. 2:**
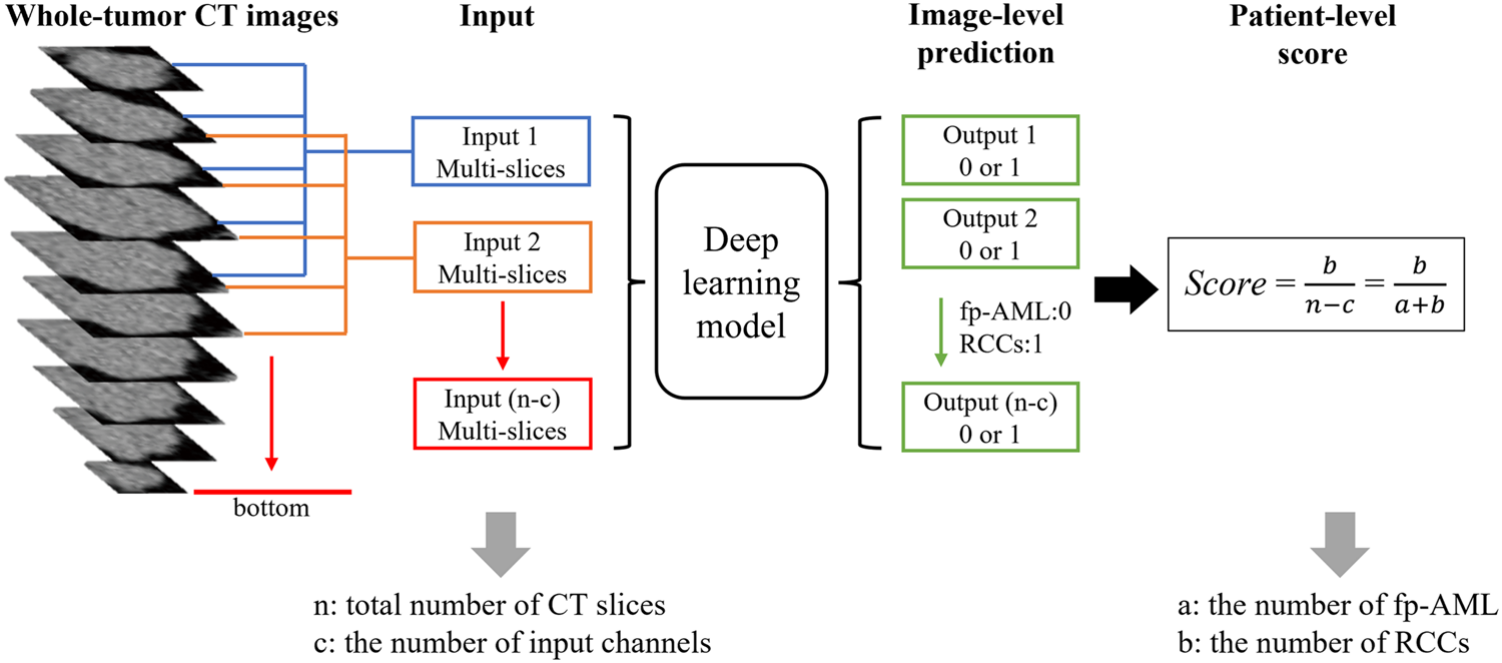
Overview of whole-tumor CT images processed by the deep learning model

The input to the CNN model was composed of single or multiple consecutive CT slices from whole tumors depending on the number of input channels. A patient’s whole-tumor CT slices were processed by the model to obtain multiple image-level predictions. Using these image-level predictions, we calculated a patient-level score for each patient, as shown in Fig. 2. The patient-level scores helped us to divide patients into two defined clinical categories: fp-AML and RCC. The optimal classification threshold was determined by receiver operating characteristic (ROC) curve analysis in a manner that maximized the Youden index.

The performance of the model was evaluated at the image level and the patient level. The categorical accuracy (ACC) metric was used to evaluate the model at the image level. The area under the ROC curve (AUC), sensitivity and specificity were used to evaluate the patient-level performance of the model. In fivefold cross-validation, the average of the evaluation metrics across all five folds represents the overall performance.

All models were trained on a GeForce RTX 2080 Ti (NVIDIA) graphics processing unit and built using Python 3.7 and PyTorch 1.7. The input resolution of Xception was reduced from a matrix size of 299 × 299 to 171 × 171. Following the application of Xception, the softmax function was used to create a probability distribution over two classes; the class with the higher probability was selected as the output. The cross-entropy function was selected as the loss function. For data augmentation, we augmented the training samples by randomly flipping and scaling the images. Based on numerous preliminary experiments, training was performed using the Adam optimizer for 50 epochs, with a batch size of 120 and a learning rate of 0.01. The dropout probability before the final fully connected layer was set as 0.2. In each batch of training, CT images of fp-AML were dynamically oversampled to strike a balance between the RCC samples and the fp-AML samples. In each fold of training, the weights were saved as the best-performing weights if the ACC performed best in the validation set.

### Independent internal validation and external validation

In the model development stage, the optimal combination of input channel number and CT modality was obtained according to the performance of the model at the patient level in cross-validation. Keeping the other hyperparameters the same as in the development phase, the model was trained using all patients in the development dataset without any tuning. Then, the model was validated with 53 unseen patients from the hold-out dataset. Furthermore, we externally validated the model using subjects from the SYSUCC cohort to evaluate the generalizability of the model. Again, we evaluated the performance of the model using ACC at the image level and the ROC curves, AUC, sensitivity, and specificity at the patient level.

### Statistical analysis

All statistical analyses were performed with SPSS software (version 22.0, SPSS, Inc., Chicago, IL, U.S.A.) and R statistical software (version 3.5.6, The R Foundation for Statistical Computing). The Pearson chi-square test or Fisher’s exact test was used to assess the distribution of categorical variables, and the independent T test was used for continuous variables. With the number of input channel as the pairing factor, the paired T test was conducted to compare the performance of the model based on enhanced CT images and unenhanced CT images in the fivefold cross-validation. The threshold for statistical significance was set as p < 0.05. In addition, 95% confidence intervals for AUC values were obtained via bootstrapping with 1000 iterations.

## Results

### Patient characteristics

This retrospective study included a total of 320 individuals from FAHSYSU and 132 individuals from SYSUCC. Patient characteristics are shown in Table 1. The proportion of patients with fp-AML, approximately 20%, was similar in both cohorts (FAHSYSU: 19%; SYSUCC: 23%). The RCC group included a mixture of ccRCC (42% vs. 44%), pRCC (19% vs. 18%) and chRCC (20% vs. 15%), with proportions well balanced amongst the two cohorts. In terms of age, sex, tumor size, location, or appearance, there were no significant differences between the two cohorts.

**Table 1:** Patient characteristics of the FAHSYSU cohort and SYSUCC cohort

Characteristic	FAHSYSU (n = 320)	SYSUCC (n = 132)	*p*
CT slices, n	26,256	10,403	
Subtype, n (%)			0.454
fp-AML	60 (19)	31 (23)	
ccRCC	135 (42)	58 (44)	
pRCC	62 (19)	24 (18)	
chRCC	63 (20)	19 (15)	
Age, y, mean ± SD	51 ± 15.8	51 ± 11.4	0.810
Sex, n (%)			0.542
M	184 (58)	80 (61)	
F	136 (42)	52 (39)	
Maximum tumor diameter, mm, mean ± SD	36.1 ± 15.8	38.6 ± 18.1	0.16
Location, n (%)			0.388
Left	167 (52)	63 (48)	
Right	153 (48)	69 (52)	
Appearance, n (%)			0.177
Exophytic	294 (92)	126 (95)	
Endophytic	26 (8)	6 (5)	

*FAHSYSU* First Affiliated Hospital of Sun Yat-Sen University, *SYSUCC* Sun Yat-Sen University Cancer Center, *SD* standard deviation, *fp-AML* fat-poor angiomyolipoma, *ccRCC* clear cell renal cell carcinoma, *pRCC* papillary renal cell carcinoma, *chRCC* chromophobe renal cell carcinoma

### Development phase and cross-validation

**Fig. 3:**
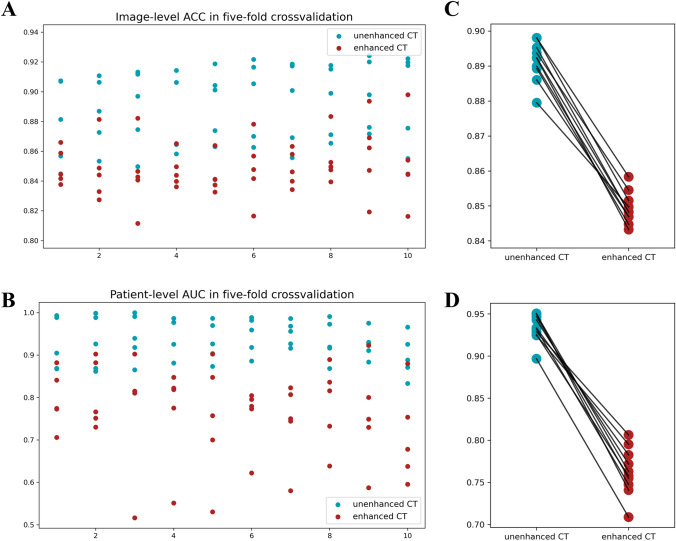
The performance of models trained on different CT modalities in the fivefold cross-validation. Scatter plots of the model's the image-level (**a**) and patient-level (**b**) performance in each fold. The paired T test of the mean values of image-level ACC (**c**) and patient-level AUC (**d**)

Table 2 shows the fivefold cross-validation results of the 20 combinations in the model development phase. As shown in Fig. 3a, b, scatter plots were used to show the model's performance at the image-level and patient-level in each fold. The paired T test was used to compare the mean values of image-level ACC and patient-level AUC of models trained on different CT modalities in the fivefold cross-validation (Fig. 3c, d). Overall, the models trained on unenhanced CT images performed better than those trained on enhanced CT images, both at the image level (p < 0.001) and at the patient level (p < 0.001).

**Table 2:** The cross-validation results of 20 models with different combinations

CT modality	Number of input channel	Evaluation metrics			
		Image-level ACC^a^	Patient-level		
			Sensitivity^a^	Specificity^a^	AUC^a^
Unenhanced CT	1c	0.879 ± 0.026	0.949 ± 0.017	0.880 ± 0.117	0.924 ± 0.056
	2c	0.886 ± 0.021	0.963 ± 0.024	0.880 ± 0.075	0.929 ± 0.058
	3c	0.889 ± 0.024	0.968 ± 0.018	0.900 ± 0.089	0.943 ± 0.049
	4c	0.890 ± 0.024	0.917 ± 0.048	0.940 ± 0.080	0.949 ± 0.040
	5c	0.892 ± 0.021	0.931 ± 0.026	0.900 ± 0.063	0.932 ± 0.042
	6c	0.895 ± 0.024	0.945 ± 0.037	0.920 ± 0.075	0.947 ± 0.039
	7c	0.892 ± 0.026	0.903 ± 0.026	0.960 ± 0.049	0.951 ± 0.026
	8c	0.894 ± 0.022	0.922 ± 0.028	0.900 ± 0.089	0.933 ± 0.044
	9c	0.898 ± 0.022	0.940 ± 0.043	0.860 ± 0.049	0.925 ± 0.030
	10c	0.898 ± 0.027	0.954 ± 0.033	0.840 ± 0.102	0.897 ± 0.046
Enhanced CT	1c	0.850 ± 0.011	0.880 ± 0.058	0.680 ± 0.117	0.795 ± 0.061
	2c	0.847 ± 0.019	0.802 ± 0.115	0.800 ± 0.063	0.806 ± 0.071
	3c	0.845 ± 0.022	0.785 ± 0.125	0.740 ± 0.224	0.772 ± 0.132
	4c	0.847 ± 0.010	0.843 ± 0.097	0.700 ± 0.167	0.763 ± 0.108
	5c	0.843 ± 0.011	0.908 ± 0.052	0.620 ± 0.204	0.747 ± 0.129
	6c	0.848 ± 0.020	0.830 ± 0.057	0.680 ± 0.147	0.755 ± 0.067
	7c	0.848 ± 0.011	0.889 ± 0.054	0.640 ± 0.120	0.741 ± 0.086
	8c	0.855 ± 0.015	0.908 ± 0.077	0.660 ± 0.150	0.783 ± 0.088
	9c	0.858 ± 0.025	0.885 ± 0.060	0.660 ± 0.174	0.758 ± 0.109
	10c	0.851 ± 0.026	0.940 ± 0.052	0.520 ± 0.117	0.709 ± 0.100

^a^The data are reported as the mean ± SD based on fivefold cross-validation

*SD* standard deviation, *ACC* accuracy, *AUC* area under the receiver operating characteristic curve

In terms of image-level performance, the worst ACC of the unenhanced CT models was 0.886 ± 0.021, while the best ACC of the enhanced CT models was only 0.858 ± 0.025. In the unenhanced CT models, ACC increased with the increase in the number of input channels, while ACC was unstable in the enhanced CT models.

In terms of patient-level performance, the unenhanced CT models also outperformed the enhanced CT models, especially in terms of AUC and specificity. The lowest AUC of the unenhanced CT models was 0.897 ± 0.046, while the highest AUC of the enhanced CT models was 0.806 ± 0.071. Among all model combinations, the “unenhanced CT and 7-channel” model achieved the best AUC of 0.951 ± 0.026 with a sensitivity of 0.903 ± 0.026 and a specificity of 0.960 ± 0.049. Based on the AUC performance, we finally selected the “unenhanced CT and 7-channel” model as the optimal combination of our multichannel deep learning model for independent internal and external validation.

### Independent internal validation and external validation

**Fig. 4:**
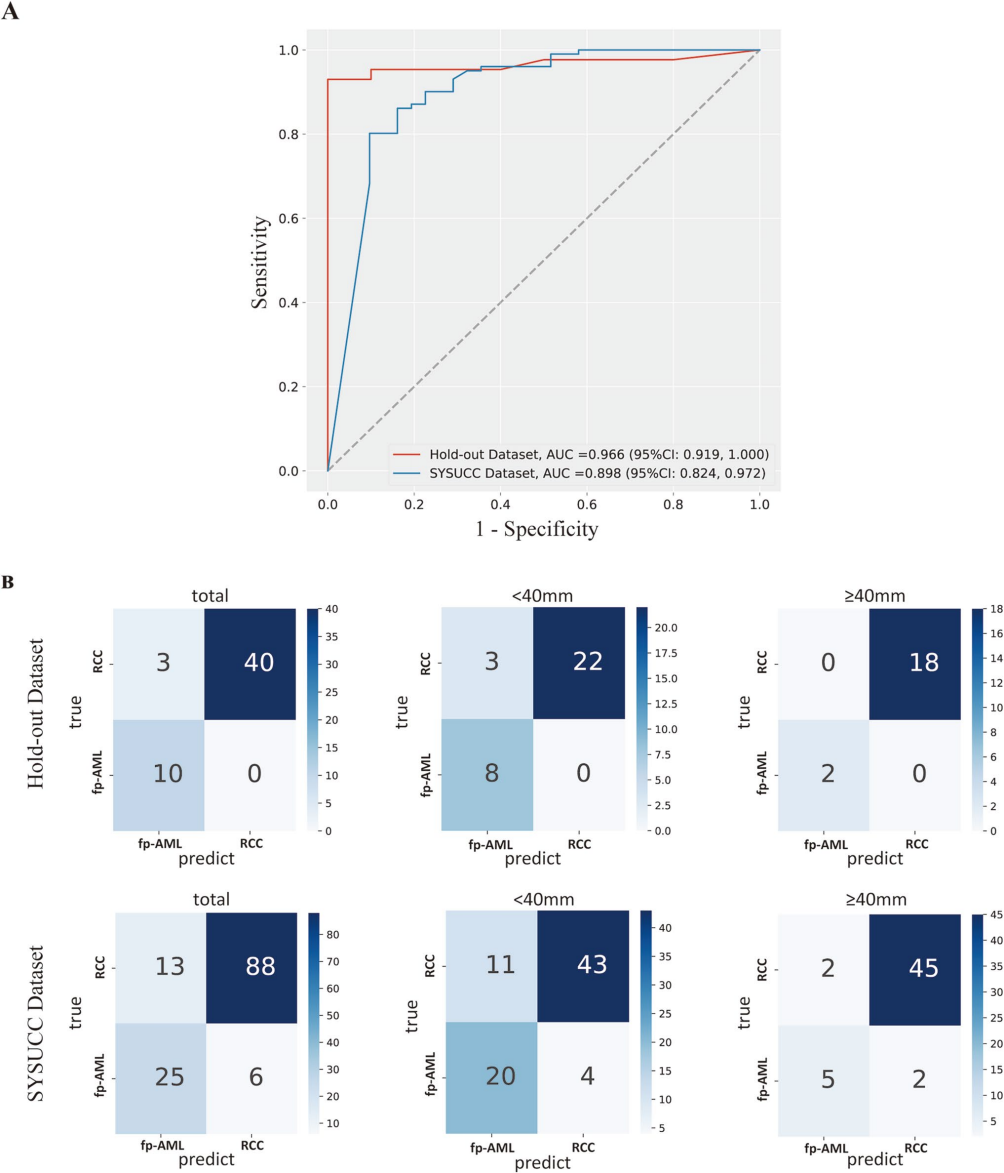
Comparison of performance of the “unenhanced CT and 7-channel” model in independent internal and external validation. **a** The receiver operating characteristic (ROC) curves for patient-level performance on the hold-out dataset and Sun Yat-Sen University Cancer Center (SYSUCC) dataset. **b** The confusion matrix for the independent internal validation and external validation

In independent internal validation (hold-out dataset), the image-level ACC of the “unenhanced CT and 7-channel” model was 0.921. At the patient level, the AUC reached 0.966 [95% confidence interval (CI) 0.919–1.000] with a sensitivity of 0.930 and a specificity of 1.000. In external validation (SYSUCC dataset), the image-level ACC was 0.865. At the patient level, the AUC was 0.898 (95% CI 0.824–0.972), with a sensitivity of 0.802 and a specificity of 0.903 (Fig. 4a). Compared with internal validation, both the image-level performance and the patient-level performance of the model decreased in external validation.

Furthermore, we compared the performance of the model when the maximum tumor diameter was < 40 mm and ≥ 40 mm. As shown in Table 3, the “unenhanced CT and 7-channel” model performed better when the maximum tumor diameter was ≥ 40 mm in both internal validation (AUC 1.000 [95% CI 1.000–1.000] vs. 0.942 [95% CI 0.863–1.000]) and external validation (AUC, 0.973 [95% CI 0.932–1.000] vs. 0.873 [95% CI 0.776–0.970]). The confusion matrix for the independent internal validation and external validation is shown in Fig. 4b.

**Table 3:** Performance of the “unenhanced CT and 7-channel” model on the hold-out and SYSUCC datasets

Dataset	Image-level ACC	Patient-level		
		Sensitivity^a^	Specificity^a^	AUC^b^
Hold-out dataset	0.921	0.930 (40/43)	1.000 (10/10)	0.966 (0.919, 1.000)
< 40 mm	–	0.880 (22/25)	1.000 (8/8)	0.942 (0.863, 1.000)
≥ 40 mm	–	1.000 (18/18)	1.000 (2/2)	1.000 (1.000, 1.000)
SYSUCC dataset	0.865	0.871 (88/101)	0.807 (25/31)	0.898 (0.824, 0.972)
< 40 mm	–	0.796 (43/54)	0.833 (20/24)	0.873 (0.776, 0.970)
≥ 40 mm	–	0.957 (45/47)	0.714 (5/7)	0.973 (0.932, 1.000)

^a^The data in parentheses are the numbers of patients

^b^The data in parentheses are 95% confidence interval

*SYSUCC* Sun Yat-Sen University Cancer Center, *ACC* accuracy, *AUC* area under the receiver operating characteristic curve

**Fig. 5:**
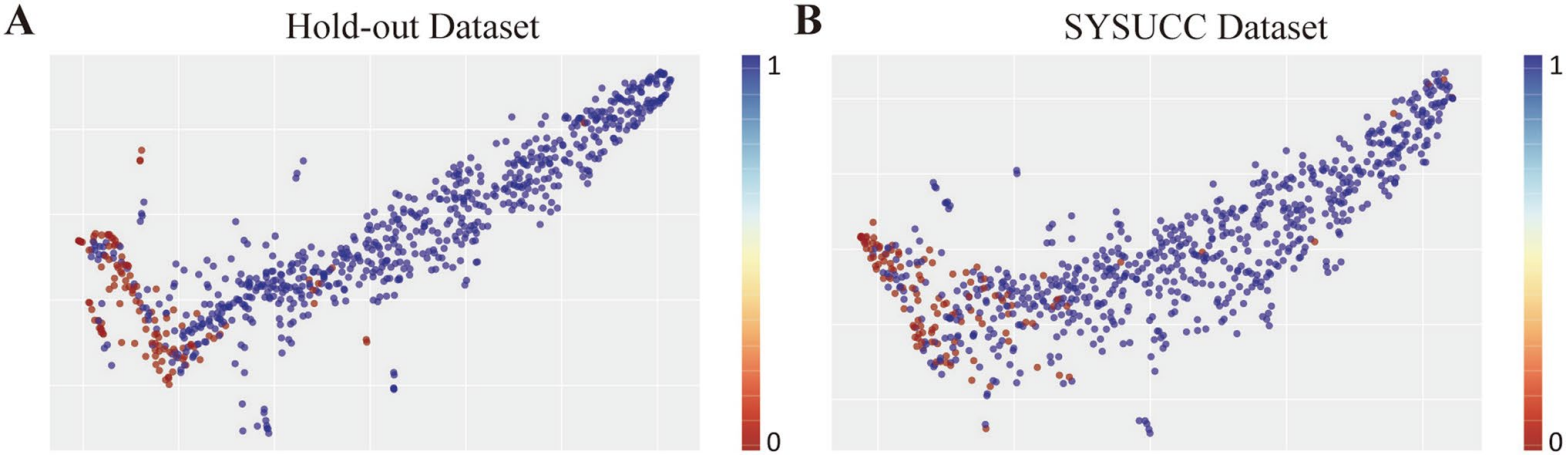
The t-SNE visualization was performed with image-level samples randomly selected from the hold-out dataset (**a**) and SYSUCC dataset (**b**). The red dots representing fat-poor angiomyolipoma (fp-AML) are mainly concentrated in the lower left of the coordinate system, and the blue dots representing renal cell carcinoma (RCC) are in a cord-like distribution on the right side

**Fig. 6:**
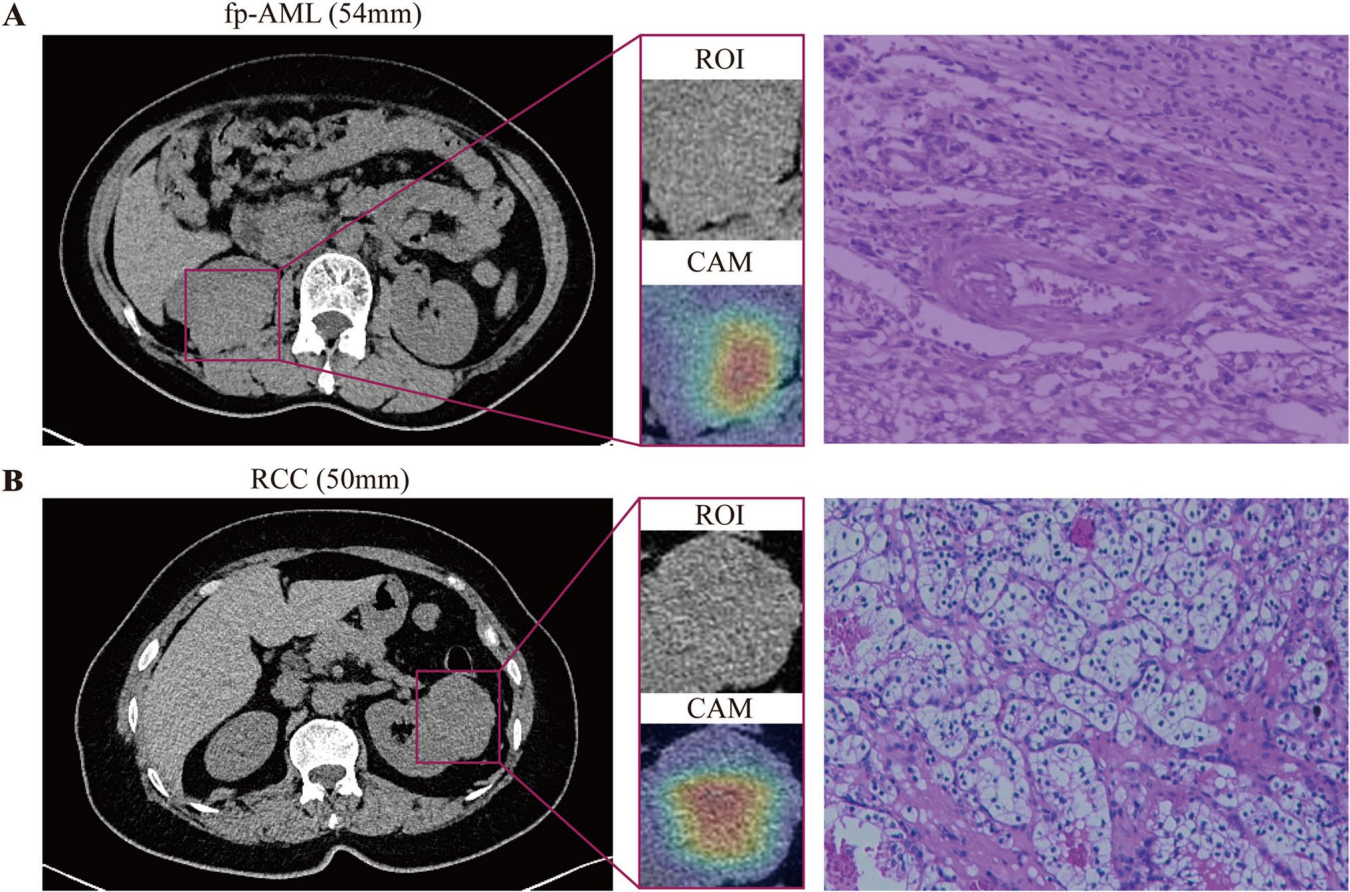
Representative example predictions from Sun Yat-Sen University Cancer Center (SYSUCC). **a** An unenhanced CT image from a 50-year-old woman who was preoperatively diagnosed with renal carcinoma but eventually confirmed by pathology as fat-poor angiomyolipoma (fp-AML). Due to a misdiagnosis of the tumor, she underwent a radical nephrectomy and lost the chance to preserve her right kidney. Our model successfully identified this tumor as fp-AML. **b** An unenhanced CT image from a 54-year-old woman who underwent a partial nephrectomy and was pathologically confirmed as clear cell renal cell carcinoma (ccRCC). Our model successfully identified this tumor as renal cell carcinoma (RCC). *ROI* region of interest, *CAM* class activation mapping

In addition, we applied t-distributed stochastic neighbor embedding (t-SNE) to visualize the basis of classification. The t-SNE visualization showed that the dots in the SYSUCC dataset were significantly more scattered than those in the hold-out dataset, which was consistent with the performance of the model on the image-level ACC (Fig. 5a, b). Furthermore, one case of fp-AML (Fig. 6a) and one case of RCC (Fig. 6b) from the SYSUCC dataset were selected to demonstrate the diagnostic performance of the model. According to the class activation mapping (CAM) images of the ROI, the region of high predictive value was located in the center of the tumor, whether fp-AML or RCC. This indicates that information about the central region of the tumor was critical for the model to discriminate between fp-AML and RCC.

## Discussion

In this study, we collected CT data from 91 patients with fp-AML and 361 patients with RCC from two centers. The multichannel deep learning model based on whole-tumor unenhanced CT images developed in our study achieved an AUC of 0.966 (95% CI 0.919–1.000) in independent internal validation. We further evaluated the generalization performance of the model with an external dataset and obtained an AUC of 0.898 (95% CI 0.824–0.972). Moreover, our model performed better with large tumors (≥ 40 mm) than with small tumors (< 40 mm) in both internal and external validation. This indicates that larger tumors can provide more CT slices and thus provide more useful information to our multichannel deep learning model.

To our best knowledge, our study enrolled a larger number of patients and achieved higher accuracy than any previous study (Hodgdon et al. 2015; Lee et al. 2017, 2018; Feng et al. 2018; Cui et al. 2019; Yang et al. 2020). Also, the present study is the first to evaluate the generalizability of the model using a dataset from an external center. In terms of algorithms, deep learning was used in this study, while machine learning was used in previous studies. The use of end-to-end training and prediction removes the need for deep learning algorithms, such as CNNs, to involve burdensome feature engineering. These engineering features are mainly hidden in numerous layers of a CNN, and learned from data using a general-purpose learning procedure. Another advantage of deep learning is its ability to better fit large datasets. Therefore, facing the growing amount of medical data, deep learning has great potential.

It is noteworthy that unenhanced CT images were more suitable for distinguishing fp-AML from RCC than enhanced CT images, according to the results of fivefold cross-validation in the development phase. Compared with tri-phase enhanced scans, the measurements acquired from unenhanced scans are more stable. The quality of enhancement CT imaging is often affected by a variety of factors, including renal function, the concentration of the contrast medium, and the scanning protocol used. Collectively, these factors make it difficult to reproduce results derived from enhancement measurements and have even led to different conclusions in the earlier literature (Kim et al. 2004; Zhang et al. 2007; Yang et al. 2013).

In practice, it is noticed that radiologists always need to consider the continuity between adjacent slices when analyzing CT images. The multichannel CNN was designed to simulate the behavior of radiologists reading CT images. By increasing the number of input channels of the CNN, we can input multiple continuous CT images into the model at the same time. Several studies have confirmed that deep learning models based on multi-slice CT images perform better than those based on single-slice CT images (Zhang et al. 2018; La Greca Saint-Esteven et al. 2022; Takao et al. 2022). Our study also confirms the correctness of the above view that appropriately increasing the number of input channels can improve the performance of the model. This may benefit from the structure of the multichannel model, which allows us to deliver more effective information to the CNN in one input. However, as the number of input channels increases, the computational load of the model also increases. Therefore, the number of input channels should be within a reasonable range to avoid excessive computational load.

Furthermore, whole-tumor CT images were used to train the model in this study. In some early CT image-based AI studies (Yan et al. 2015; Feng et al. 2018), only one or several representative CT slices of each tumor were used for feature extraction and model training. The process of CT slice selection is clearly subjective and could lead to instability. Critical information may be missed in the selected slices, thus affecting the performance of the model. Here, the use of whole-tumor CT slices allowed us to avoid such problems while also making our dataset suitable for the training needs of multichannel CNN.

The performance of our model decreased in the external validation compared to the internal validation in this study. In our opinion, both the hardware and software of the CT scanner have an impact on model performance. Model performance may also be affected by different CT scanning protocols. What's more, the number of samples may not be sufficiently representative of the whole population, especially in the case of rare categories. As there may be other reasons for the degradation of the model performance in the external dataset, this is a further interesting and worthwhile topic to be investigated.

While promising results have been obtained from our multichannel deep learning model, there do exist several limitations. Firstly, CT images of the nephrographic and excretory phase were not used for model training in this study. This is mainly due to the long-time span of cases that we reviewed. In most of the early cases, the image quality of nephrographic and excretory phase is far inferior to that of the corticomedullary phase in terms of the thickness of the reconstruction, tumor scan integrity, and scan timing. Secondly, the use of whole-tumor CT slices can make the labeling of ROIs burdensome. Developing an accurate and stable automatic segmentation method for tumor ROIs will help to improve the practicability of our classification model. Moreover, a larger multi-center validation study will be needed to further assess the robustness of the model across populations. Also, given the retrospective nature of this study, a prospective study may be required to evaluate the clinical value of our model.

In conclusion, the present study demonstrated that a multichannel deep learning model based on whole-tumor unenhanced CT images represents a highly useful tool for differentiating fp-AML from RCC. This tool may improve the accuracy of preoperative diagnosis for patients with renal masses and therefore facilitate the clinical decision-making process.

## Data Availability

The CT imaging data and clinical information in the current study are not publicly available due to patient privacy obligations but are available from the corresponding authors on reasonable request.
